# Correlation between maternal and childhood VitB12, folic acid and ferritin levels

**DOI:** 10.12669/pjms.331.10998

**Published:** 2017

**Authors:** Fatima Zeeshan, Attia Bari, Saima Farhan, Uzma Jabeen, Ahsan Waheed Rathore

**Affiliations:** 1 Fatima Zeeshan, MRCPCH, FCPS. Department of Paediatric Medicine, The Children’s Hospital and The Institute of Child Health, Lahore, Pakistan; 2 Attia Bari, DCH, MCPS, FCPS. Department of Paediatric Medicine, The Children’s Hospital and The Institute of Child Health, Lahore, Pakistan; 3 Saima Farhan, FCPS. Department of Microbiology, The Children’s Hospital and The Institute of Child Health, Lahore, Pakistan; 4 Uzma Jabeen, FCPS. Department of Paediatric Medicine, The Children’s Hospital and The Institute of Child Health, Lahore, Pakistan; 5 Ahsan Waheed Rathore, MRCP, MRCPCH, FRCP. Department of Paediatric Medicine, The Children’s Hospital and The Institute of Child Health, Lahore, Pakistan

**Keywords:** Anemia, Vit B12 deficiency anemia, Folic acid deficiency

## Abstract

**Objective:**

To determine the correlation between serum folic acid, vitamin B12 and ferritin of mother and child and to study various neonatal risk factors as a cause of anemia in children.

**Methods:**

One hundred eighty children two months to two years of age admitted in the department of Pediatric Medicine of The Children’s Hospital and The Institute of Child Health Lahore from January 2013 to January 2015 with common medical conditions having anemia were included. Complete blood count (CBC), serum ferritin level, folic acid and Vitamin (Vit) B12 level were sent of children and their mothers. Data was analyzed using SPSS version 20.

**Results:**

Out of 180 children with anemia, 66.7% were males. Mean age of children was 7.3months. Fifty-five percent children were malnourished according to z scoring. The mean Hemoglobin (Hb) of children was 8 g/dl. Only 4% children had low ferritin level while 60% had low folic acid and 45% had decreased VitB12. There was significant correlation between Hb of mother and child (p =0.02), Vit B12 deficiency (p=0.008) and iron deficiency (p<0.001). Premature children had lower folic acid levels (p =0.02), while prematurity, IUGR, previous admission and history of sepsis showed no association with anemia in our study. Both breast-feeding and top feeding showed significant association with anemia with p-value of 0.042 and 0.003 respectively while dilution showed no impact on anemia.

**Conclusion:**

Maternal anemia has a significant impact on child’s hemoglobin. As compared to previous concept of increased iron deficiency in children we found increased occurrence of folic acid and VitB12 deficiency in children and their mothers.

## INTRODUCTION

Anemia is one of the commonest public health problems.^1^ More than 61% of Pakistani children under five are anemic according to WHO survey conducted in 2011-2012. There is a high prevalence of anemia affecting women of child bearing age and preschool children. Iron deficiency is responsible for most of the cases.^2^ Nutritional megaloblastic anemia occurs commonly among under-nourished societies of tropical and subtropical countries and incidence has dramatically increased over pastdecades.^1^,^2^ Apart from iron deficiency other nutritional deficiencies in the mother needs to be addressed to decrease childhood anemia.^3^

Anemia not only leads to maternal morbidity but also affects perinatal outcome. The youngest are the worst affected as a study shows that iron deficiency is most prevalent in areas of poor access where mothers are also deficient.^4^ It is imperative to address anemia in mothers to prevent its occurrence in children. The link of iron deficiency between mother and child is supported in analysis of nutrition survey.^4^ However, previously there has been no detailed study in Pakistan of the biochemical etiology of this anemia, exploring the relative contributions of iron, vitamin B12 and folate deficiency. This paper considers child and maternal Hb, iron, folate and B12 status, with possible implications for both maternal and infant health. It also takes into account history of low birth weight, prematurity, sepsis and prior hospitalization in anemic children to find any association of anemia with these risk factors.

The objective of this study was to determine the correlation of hemoglobin, ferritin, VitB12 and folic acid between mother and children and to study different neonatal risk factors as a cause of childhood anemia.

## METHODS

This was a prospective hospital based study conducted over a period of two years from January 2013 to January 2015. We enrolled 180 children two months to two years of age along with their 180 mothers. Children with common medical problems having hemoglobin (Hb) <10 g/dl admitted in General medical ward of The Children’s Hospital Lahore were included. We excluded children who received a blood transfusion, those having any known cause of anemia like hemolytic anemia, thalassemia, worm infestation, malignancy and those on iron, folic acid or Vit B12 supplements. Informed consent was taken from mothers. Anemia was defined in children and mothers as Hb<10g/dl. Mild anemia in children was defined as Hb from7-9.9g/dl, moderate anemia as Hb 5.1-7g/dl and severe anemia was as Hb ≤5g/dl. Using WHO criteria iron deficiency in mother and child was categorized by anemia with serum ferritin level < 12ng/ml.^5^ Using the manufacturer’s reference ranges folic acid deficiency in mother and child was defined as anemia with folic acid <4ng/ml while VitB12 deficiency in mother and child was defined as anemia with level of Vit B12<210pg/ml.

The ethical approval was taken from local hospital ethical committee. Informed consent was taken from mothers by their signatures or thumb impression in case of those who were illiterate. Weight and height was assessed by WHO z scoring and the neonatal history of prematurity, low birth weight, previous admission and sepsis was sought. Five ml blood was drawn from both mother & child. Complete blood count, ferritin, folate, vitamin B12 levels were performed using Immulite 2000 based on radioimmunoassay technique.

The data was analyzed using Statistical Package for Social Sciences (SPSS) version 20 software. Variables were summarized using frequencies and percentages for categorical variables, and median, and range for continuous variables. The chi-square test was used for statistical analysis of categorical variables. A p value of <0.05 was considered statistically significant.

## RESULTS

One hundred eighty children with anemia were enrolled into the study. Out of these 120 (66.7%) were males and 60 (33.3%) were females with Male: Female ratio was 2:1. The mean age of children was 7.3 ±5.6 months. Nearly two third, 132 (73.3%) children were under one year while 23.3% were above one year. The distribution of anemia according to severity is shown in Table-I. Forty-five percent children in our study had normal weight while 55% were malnourished as shown in Table-II according to WHO z scoring. Fifty-three percent children had complete vaccination while 47% had incomplete vaccination. Forty-three percent were breast-fed, 20% were on bottle-feeding, out of which 79% were using diluted milk and 37% were both top plus breast-fed. The mean Hb of children was 8 g/dl ±1.4g/dl. In our data only 7 (4%) children had ferritin less than 12ng/ml, while 75% had low folic acid levels and 64% had decreased VitB12 levels. Mean maternal age was 26.5 ± 5.5 years. Hemoglobin level of 61 (34%) mothers was normal while 119 (66%) were anemic. Mean maternal Hb was10.4 ± 1.45g/dl.

**Table-I:** Distribution of anemia in children according to severity.

Categories of anemia	Hemoglobin level	No. (Percentage) n=180
Mild	7.1-10 g/dl	147 (82%)
Moderate	5-7 g/dl	27 (15%)
Severe	< 5 g/dl	6 (3%)

**Table-II:** Demographic data.

Parameter		Frequency n=180	Percentage
Gender	Female	120	66.7%
	Male	60	33.3%
Maturity	Term	153	96.7%
	Preterm	27	3.3%
IUGR	Yes	6	3.3%
	No	174	96.7%
Diagnosis	Acute Watery Diarrhea	52	28.9%
	Bronchopneumonia	55	30.6%
	Febrile Fits	7	3.9%
	Meningitis	44	24.4%
	Sepsis	16	8.9%
	Asthma	6	3.3%
Z-scoring	Normal	81	45.%
	-1to--2	34	18.9%
	-2 to-3	32	17.8%
	< -3	33	18%
H/o previous admission	Yes	34	19%
	No	146	81%
H/o neonatal sepsis	Yes	4	2.2%
	No	176	98%

Premature children had lower folic acid levels (p =0.02), while prematurity, (intrauterine growth retardation) IUGR, previous admission and history of sepsis showed no association with anemia in our study. Both breast-feeding and top feeding showed significant association with anemia with p-value of 0.042 and 0.003 respectively while dilution showed no impact on anemia(p=0.2) in our study. Intrauterine growth retarded babies and exclusive breast feeding was also linked to anemia in mothers (Table-III).

**Table-III:** Relationship of maternal and child anemia.

Parameter	p value
Hemoglobin of mother and child	0.02
Ferritin of mother and child	<0.001
VitB12 of mother and child	0.008
Folic acid of mother and child	0.8
Intrauterine growth retardation with mother’s Hb	0.02
Exclusive breast feeding with mother’s Hb	0.05

**Fig. 1:**
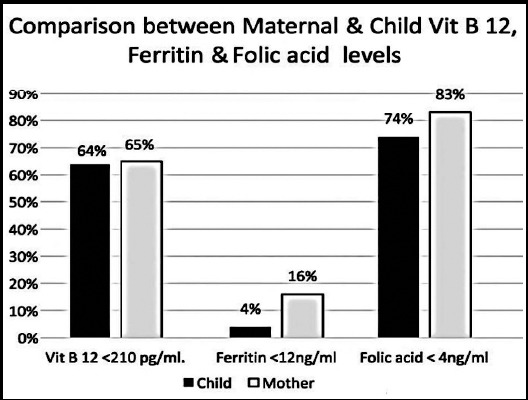
Maternal and child anemia comparison.

Ferritin deficiency was found in 15.6% mothers whereas 83% were folic acid deficient. Vit B12, folic acid and ferritin correlation shown in Fig.1. The relationship between different lab and clinical parameters between mother and child are shown in Table-III. The relationship between Hb of mother and child was significantly associated (p =0.02). The children with low ferritin values also belonged to iron deficient mothers (p <0.001). Mothers who had Vit B12 deficiency also had children with low Vit B12 levels (p=0.008) while there was no significant correlation observed between maternal and child folic acid levels (p=0.8).

## DISCUSSION

Anemia is globally the most common nutritional disorder especially in resource limited countries. The prevalence of anemia is 47% among rural and 26% among urban females aged 15 to 44 years in Pakistan.^5^ The incidence of anemia in children reaches to 60% in Africa.^6^ Anemia is particularly common in under two as it reflects maternal stores. There are many causes of which most common are nutritional, folic acid, Vit B12 deficiency and worm infestation.^7^ Our study shows that maternal anemia has a significant impact on child’s hemoglobin. Along with iron deficiency VitB12 is strongly linked between them. We found no correlation between maturity, birth weight and previous sepsis with anemia. In a study conducted in Pakistan, maternal anemia was linked with low birth weight.^8^ There are studies which suggest links of prematurity with anemia.^7^ In another study in Tanzania in 2015 there was also no association observed between birth weight and anemia.^8^

In our data 55% children were malnourished with 18% having severe malnutrition. This is important as malnutrition can lead to increased morbidity and mortality with anemia.^9^ No significant relation was found between z scores and anemia. This can be due to the release of iron from reduced red cell mass in anemia which is then stored as ferritin so the underweight children may not have iron deficiency anemia. 9 Low maternal folic acid status has also been linked with low birth weight^2^ but in this study we had only 3% low birth weight babies (Table-II) so relationship could not be determined.

We found both breast feeding and top feeding associated with anemia. Previous studies show iron deficiency anemia with prolonged exclusive breast feeding.^10^ In another study in China breast fed babies had low Hb than formula feed which points to the need of iron fortification for breastfed babies^11^ and early interruption of breast feeding as also been linked with iron deficiency anemia.^7^ Maternal Hb is associated with exclusively breast feeding in another study.^12^ In our study top fed children had significant association with Vit B12 deficiency anemia (p= 0.006) but we did not ask about weaning and use of vegetable based nutrition.

In our study 64% children were Vit B12 deficient with significant correlation (p =0.02)with mother. Infants born to Vit B12 deficient mothers have stores to sustain at least four months but those on breast feed may get deficiency if mothers are vegetarian.^2^,^13^ Literature shows 3% children under four having Vit B12 insufficiency.^14^ A recent survey in Canada showed that one in twenty women suffer from Vit B12 deficiency and another study showed that less than 50% had desired levels. There is not much data on Vit B12 deficiency in children. It is important as Vit B12 deficiency leads to neurological impairment.^15^ The strong link between maternal and child’s cobalamin levels has led to birth screening programs for Vit B12 deficiency in United States.^16^

More than 50% anemia is due to iron deficiency in children under five.^17^,^18^ Iron deficiency is very common in child-bearing mothers. Nearly half of women are at risk of developing anemia in pregnancy.^7^ In our study 29% of women were iron deficient and only 7 (4%) children had low ferritin but their mothers were also iron deficient (p value 0.02). This could be due to the reason that all our children were admitted due to some common medical condition, so ferritin being acute phase reactant could have increased. Another study in UK showed 40 percent of women (19-34years) have low iron levels. A study in India showed increased ferritin threshold for children with increased C reactive protein.^3^ Insufficient iron can affect brain myelination.^7^ Iron deficiency has been linked with prematurity and low birthweight.^19^ We did not find this association which can be due to the small number of preterm and low birth weight babies (Table-II).

Low folate levels in pregnant mothers affect the folate status of children.^15^ In our study 83% of women were folic acid deficient while 75% of children had folate deficiency although we did not see significant correlation. The low folate leads to low birth weight and developmental problems. We found association of folic acid deficiency with prematurity in our study (p=0.02) which is similar to finding in a study conducted over 2000 pregnant females.^2^ We did not look for any neural tube defects in these children. Low folic acid levels were significantly correlated with low Vit B12 levels and their interrelationship has been documented.^20^ Vit B12 can be low in infants secondary to mother levels and manifests itself from 2-12 months as failure to thrive with sometimes faltering head centiles and neuro-developmental delay.

This highlights the importance of ensuring that women have excellent nutritional status prior to conception hence the emphasis should be shifted from antenatal care to pre-pregnancy health especially in developing countries where there are multiple pregnancies in young age and limited access to standard health care.^4^,^21^

## CONCLUSION

Maternal anemia has a significant impact on child’s hemoglobin. Along with iron deficiency, folic acid and Vit B12 deficiencies are also a major contributor to both child’s and maternal anemia. Maternal and child ferritin and VitB12 levels are strongly correlated. It is imperative to improve general health and hemoglobin of women at childbearing age to protect their children from anemia and its consequences.
